# Ecobody technology: rapid monoclonal antibody screening method from single B cells using cell-free protein synthesis for antigen-binding fragment formation

**DOI:** 10.1038/s41598-017-14277-0

**Published:** 2017-10-25

**Authors:** Teruyo Ojima-Kato, Satomi Nagai, Hideo Nakano

**Affiliations:** Graduate School of Bioagricultural Sciences, Nagoya University, Furo-cho, Chikusa-ku, Nagoya, 464-8601 Japan

## Abstract

We report a rapid and cost-effective monoclonal antibody screening method from single animal B cells using reverse transcription (RT)-PCR and *Escherichia coli* cell-free protein synthesis (CFPS), which allows evaluation of antibodies within 2 working days. This process is named “Ecobody technology”. The method includes strategies to isolate B cells that specifically bind an antigen from the peripheral blood of immunised animals, and single-cell RT-PCR to generate DNA fragments of the V_H_ and V_L_ genes, followed by CFPS for production of fragments of antigen binding (Fab). In the CFPS step, we employed our techniques: 1) ‘Zipbody’ as a method for producing Fab, in which the association of heavy and light chains is facilitated by adhesive leucine zipper peptides fused at the C-termini of the Fab; and 2) an N-terminal SKIK peptide tag that can increase protein expression levels. Using Ecobody technology, we obtained highly-specific monoclonal antibodies for the antigens *Vibrio parahaemolyticus* and *E*. *coli* O26. The anti-*V*. *parahaemolyticus* Zipbody mAb was further produced in *E*. *coli* strain SHuffle T7 Express in inclusion bodies and refolded by a conventional method, resulting in significant antigen-binding activity (*K*_D_ = 469 pM) and productivity of 8.5 mg purified antibody/L-culture.

## Introduction

Monoclonal antibodies (mAbs) have become essential research tools and highly successful in diagnostic and therapeutic areas. Although mouse hybridoma technology has pioneered the production of mAbs^1^, and it remains an important platform both in research and industry, this method is time-consuming and restricted to particular animal species. Low fusion efficiency of a B cell and myeloma partner also limits the chance of recovery of desired mAb clones^2^. Technologies such as phage, yeast, and mammalian cell display have been widely recognised as powerful methods that increase the variety of mAbs screened from immunised or synthetic repertoires^3^. However, in most display systems, the parental libraries are constructed through random combination of heavy and light chain genes. Thus, some *in vivo* repertoires of heavy and light chains in immune responses are lost, and unnatural variable region pairs combine unproductively, resulting in few applicable antibodies^4^.

More recently, platforms have emerged that allow the direct sampling of single B cell repertories from the immune system^5^. Single B cell screening strategies, which can rapidly generate mAbs from single B cells from immunised animals, have been proven to be powerful techniques to obtain the natural antibody repertoire^6–9^. Usually in these methods, recombinant production of the mAbs is performed in transient expression systems using animal cells like CHO and HEK293, resulting in a rate-limitation of the screening process, because transfection and expression in animal cells requires at least 3–5 days^8^. In contrast, cell-free protein synthesis (CFPS) offers an alternative expression system that avoids many of the problems of conventional cell-based expression technologies^10,11^. In particular, CFPS systems have big advantages over *in vivo* methods for high-throughput recombinant protein production because the cell-free format allows for screening without requiring time-consuming gene-cloning, transformation, or cultivation^12,13^. Additionally, the process is easily modified by chemical or protein additives to improve the folding of proteins of interest^14^.

Taking advantage of CFPS systems, we developed a rapid mAb screening system named “Single-Cell Reverse Transcription-PCR linked *in vitro*-Expression (SICREX)”, which utilises animal B cells as the source and *Escherichia coli* extract-based CFPS systems to produce fragments of antigen binding (Fab), instead of animal cell-based production of whole IgG^15–17^. This method requires no transfection of DNA into living cells and no cell cultivation for protein expression *in vivo*, making the entire screening process significantly faster than methods using animal cells. Using this method, proteins can be obtained in 60 to 90 min just by mixing the *E*. *coli* cell-extract with template DNA (PCR fragments), amino acids, nucleotides, T7 RNA polymerase and an energy source. However, the SICREX system still had the following technical problems. Firstly, correct folding and assembly of the heavy chain (Hc) and light chain (Lc) of Fab were challenging in the CFPS because of intermolecular disulfide bonds, which often resulted in incorrect refolding and low assemble of Fab. In particular, active Fabs were not produced at all in the case of rabbit mAb clones, probably because of the presence of too many Cys residues involved in disulfide bond formation^18^. Therefore, reconstruction of single chain Fv (scFv) genes was required for enzyme-linked immunosorbent assay (ELISA) evaluation^17^, whereas Fabs are thought to be preferable to scFvs because of their higher binding activity and stability^19,20^. Secondly, it was difficult to obtain enough proteins in CFPS for ELISA evaluation, because the expression efficiency varied significantly depending on the gene. In some cases, optimization of the ratio of Hc and Lc gene templates included in the CFPS may be required to equalise their expression^21,22^. Thus, ELISA results in the final step of SICREX tend to lack accuracy and reproducibility, even if the mAbs obtained are excellent.

To address such problems, we have recently developed a modified Fab format named ‘Zipbody’ that contains adhesive short peptide pairs, leucine zippers (LZ) LZA and LZB or c-Jun and c-Fos, fused at the C-terminus of the Hc and Lc, respectively. We found that the fusion of the LZ to the Fab could enhance correct pairing of the Hc and Lc, leading to the production of active Fab in both *in vitro* and *in vivo E*. *coli* expression systems using several mAb clones^23^. Furthermore, we recently found that the addition of a short peptide sequence tag Ser-Lys-Ile-Lys (SKIK) to the N-terminus of so-called ‘difficult-to-express proteins’ can dramatically improve their expression level, both in *E*. *coli in vivo* and in *in vitro* expression systems^24^.

**Figure 1:**
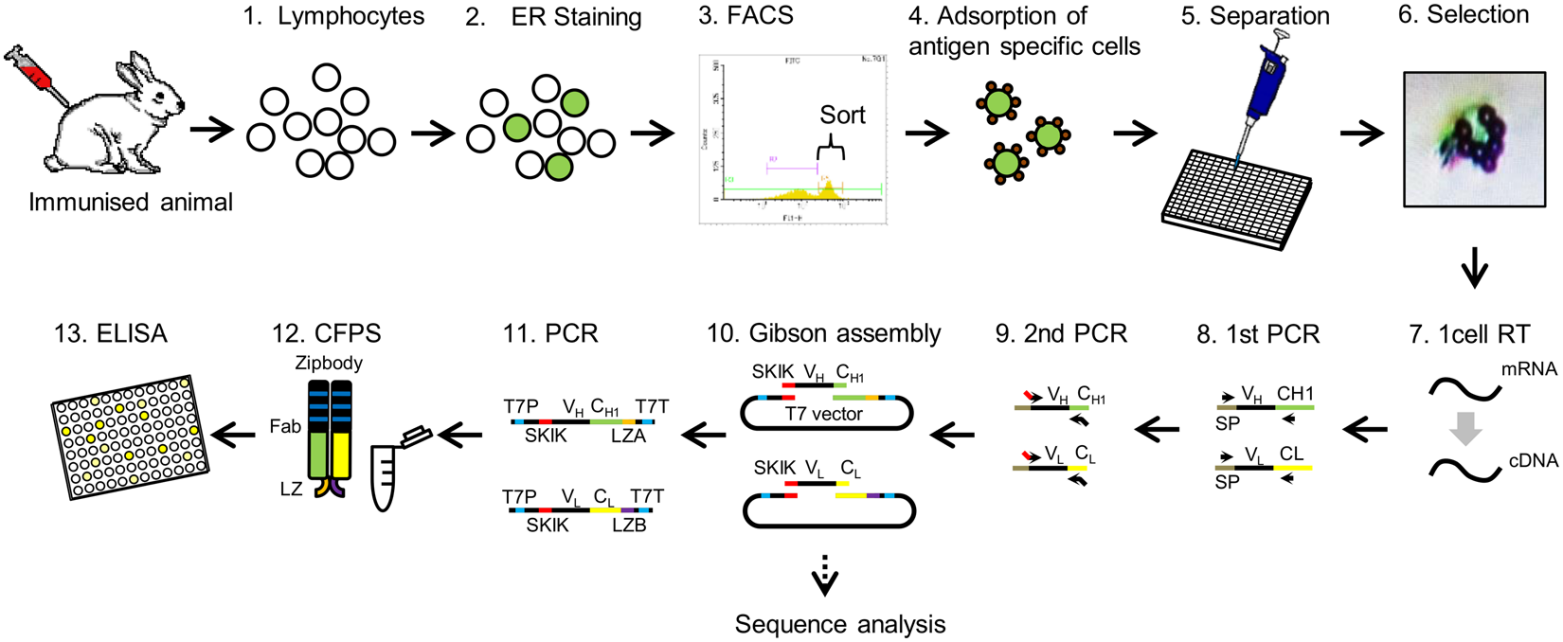
Two days protocol of Ecobody technology. SP, signal peptide sequence; T7P, T7 promoter; T7T, T7 terminator; SKIK, SKIK tag. (1) Collection of lymphocytes by density gradient centrifugation from a few millilitres of animal blood. (2) Fluorescent staining of plasma cells with endoplasmic reticulum (ER)-tracker. (3) Sorting of stained cells by fluorescence-activated cell sorting (FACS). (4) Adsorption of cells binding to the antigen using antigen-coated magnetic beads. (5) Separation of one cell per 10 µL into 384-well plate. (6) Selection and confirmation of cell-bead complexes under an inverted phase-contrast microscope. (7) Reverse transcription using gene specific primers and SuperScript IV reverse transcriptase. (8) First PCR, using primers annealing to the signal peptide (SP) sequence and constant region of antibody genes. (9) Second PCR, using primers with tails required for the subsequent Gibson assembly step. (10) Gibson assembly to combine T7 promoter and terminator with antibody genes. (11) PCR to amplify DNA fragments for cell-free protein synthesis. HA and His tags are present downstream of leucine zipper A (LZA) and B (LZB), respectively. (12) Expression of Zipbody with SKIK tag by cell-free protein synthesis (CFPS) with DsbC and oxidised glutathione (GSSG). (13) Enzyme-linked immunosorbent assay (ELISA) evaluation. DNA sequences were analysed as required using plasmids constructed in step (10) In this protocol, steps 1 to 9 are conducted for the first day and steps 10 to 13 are next day.

In this study, we describe an improved SICREX system named ‘Ecobody technology’ which combines these two significant techniques, namely Zipbody and the SKIK peptide tag, for improvement of Fab formation and protein expression in CFPS. We achieved a 2-day protocol for complete screening of antigen-specific mAbs, involving collection of B cells from peripheral blood of an immunised rabbit, selection of B cells by antigen-coated beads and endoplasmic reticulum (ER) staining, single-cell-based PCR, mAb production in CFPS, and ELISA, using the food-borne bacteria *Vibrio parahaemolyticus* and *Escherichia coli* O26 as the antigens (Fig. 1). We further describe active Zipbody production in *E*. *coli* by *in vivo* expression in inclusion bodies followed by refolding. Ecobody technology is a high-throughput and low-cost mAb screening method that has the major advantage of being combined with mass production in *E*. *coli*. Ecobody technology will be beneficial to the field of mAb research and development.

## Results

### Effect of leucine zipper fusion and SKIK tag on Fab formation in CFPS

**Figure 2:**
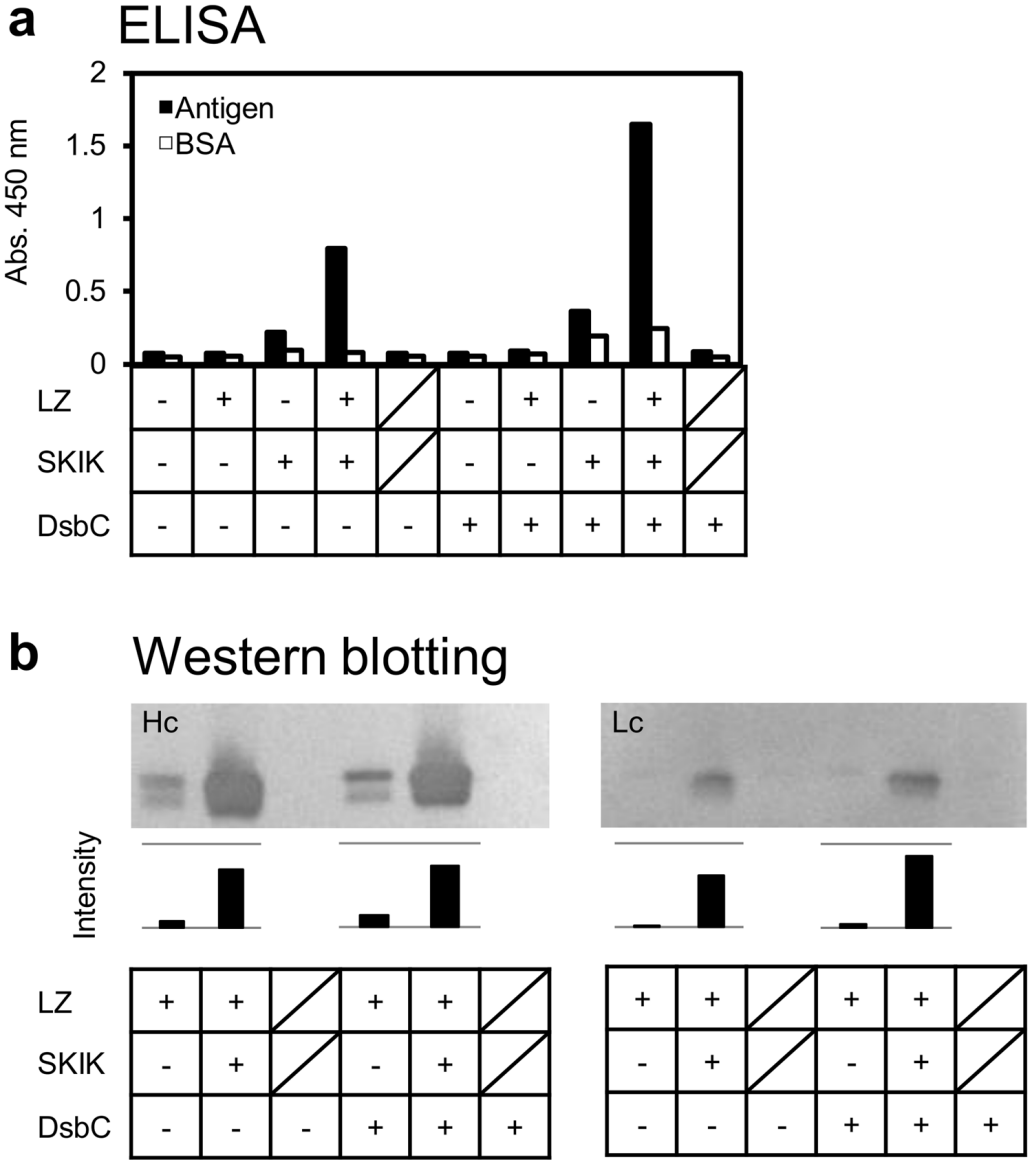
Effect of leucine zipper, SKIK tag, and DsbC on mAb synthesis in CFPS. Rabbit Fab (anti-*Listeria monocytogene*s clone) was produced by CPFS with various combinations of the presence (+) or absence (−) of the LZ fusion, N-terminal SKIK tag, and DsbC. Oblique lines indicate negative controls without DNA template. (**a**) ELISA result for *L*. *monocytogene*s antigen and bovine serum albumin (BSA; negative control). The primary and secondary antibodies were three-fold diluted CFPS samples and 2000-times diluted anti-rabbit Ig(G + M)-horseradish peroxidase (HRP) conjugate. (**b**) Western blotting of 1 µL of CPFS product separated by SDS-PAGE. Hc and Lc were visualised with anti-HA tag-HRP and anti-FLAG tag-HRP conjugated antibodies, respectively. The intensities of each band quantified by image J software are indicated at the bottom. Full-length blots are included in Supplementary Information (Supplementary Fig. S4).

First, we assessed the effect of LZ fusion, an N-terminal SKIK peptide tag, and addition of the disulfide isomerase DsbC to the CFPS using an anti-*Listeria monocytogenes* mAb clone as a model rabbit mAb (accession nos. LC030182 and LC030188^17^). We prepared four pET22b-based constructs encoding: 1) Hc-human influenza hemagglutinin (HA) tag and Lc-FLAG tag; 2) Hc-LZA-HA tag and Lc-LZB-FLAG tag; 3) SKIK-Hc-HA tag and SKIK-Lc-FLAG tag; and 4) SKIK-Hc-LZA-HA tag and SKIK-Lc-LZB-FLAG tag (Supplementary Fig. S1). The DNA fragments containing the T7 promoter and terminator were amplified from these constructs with primers F1 and R1, and used as templates for CFPS. In our previous work, this antibody did not form a functional Fab, but it worked as an scFv^17^. Here, in ELISA, slight activity was obtained when the LZ or SKIK tag was added individually, but a significantly higher ELISA signal was observed when both were added (Fig. 2a). This was contributed by an improvement in the functional pairing of the Hc and Lc mediated by the LZ fusion and improved expression with the SKIK tag fusion. In addition, it was found that the effect was more prominent in the presence of DsbC. Consistent with our previous study^24^, it was also found that addition of the SKIK tag increased protein synthesis of both the Hc and Lc in CFPS (Fig. 2b).

From the above results, we concluded that fusion of the LZ and SKIK tag to Fab and addition of DsbC to the CFPS were effective for the synthesis of active mAbs, and these steps were adopted in the following mAb screening. The combined process was named “Ecobody technology”.

### mAb screening by Ecobody technology

Figure 1 shows a flowchart of Ecobody technology developed in this study. We followed this workflow to develop mAbs that bind to two kinds of foodborne pathogenic bacteria, *V*. *parahaemolyticus* and *E*. *coli* O26.

**Figure 3:**
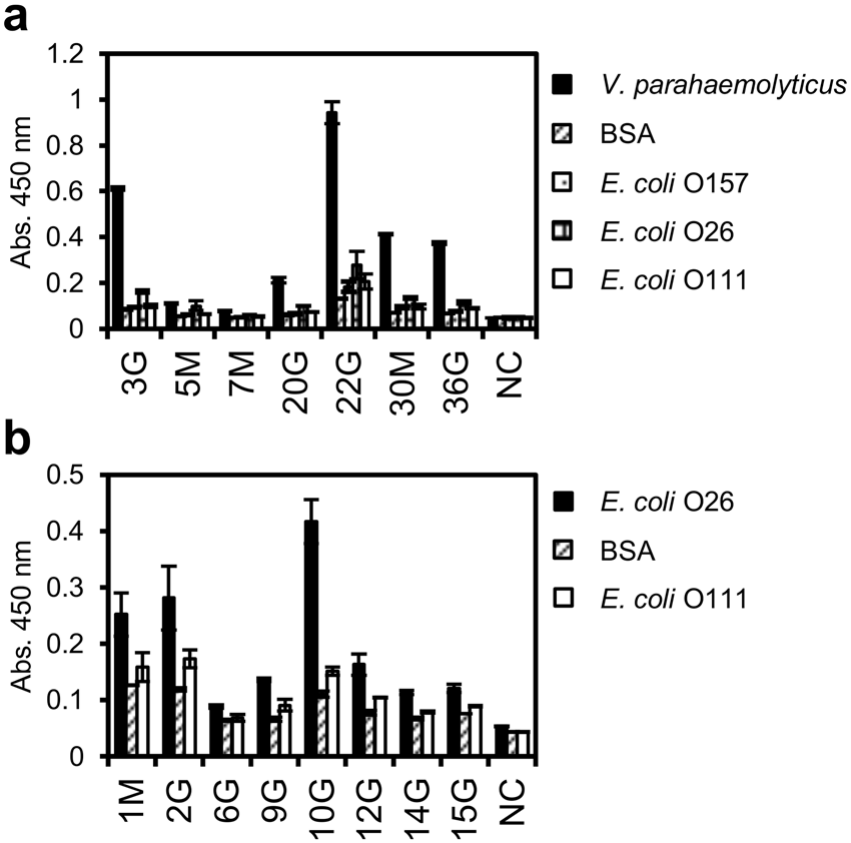
ELISA evaluation of the mAbs obtained by Ecobody technology. ELISA results for Zipbody mAbs produced in CFPS using Ecobody technology. The primary and secondary antibodies were six-fold diluted CFPS samples and 2000-times diluted anti-rabbit Ig(G + M)-HRP conjugate. NC means negative control using PBS as the primary antibody. (**a)** Seven clones with *V*. *parahaemolyticus* as the antigen. (**b)** Eight clones with *E*. *coli* O26 as the antigen.

First, 6.8 × 10^6^ lymphocytes were collected from a few millilitres of blood from a rabbit immunised with inactivated *V*. *parahaemolyticus*. The cells were stained with ER tracker and about 30% (2.0 × 10^4^ cells) showing strong fluorescence were collected by fluorescence-activated cell sorting (FACS). Subsequently, magnetic beads coated with inactivated *V*. *parahaemolyticus* were added to the cell mixture, and approximately 500 cells were separated by magnet, of which 19 were used for single-cell PCR. Seven pairs of Lc and Hc amplified gene fragments (three IgM and four IgG clones, named 3G, 5M, 7M, 20G, 22G, 30M, and 36G) were subjected to CFPS and ELISA evaluation. Protein expression from these genes was confirmed by fluorescent imaging after sodium dodecyl sulfate-polyacrylamide gel electrophoresis (SDS-PAGE) (data not shown). All the clones, expressed as the Zipbody and including the SKIK tag, had higher binding activity toward *V*. *parahaemolyticus* than toward the other tested antigens (*E*. *coli* O157, O26, O111 or BSA) (Fig. 3a). Clone 22 G had the highest ELISA signal and low cross-reactivity.

The mAb screening procedure for anti-*E*. *coli* O26 was almost the same as that described above, except for an additional step for the removal of B cells cross-reacting with *E*. *coli* O111 before selection with *E*. *coli* O26-coated beads. Starting from 3.0 × 10^6^ lymphocytes, 5.4 × 10^4^ cells were sorted by FACS and finally 750 cells were collected with *E*. *coli* O26-coated beads. From these, 22 were subjected to single-cell PCR and DNA amplifications of 10 pairs of Hc and Lc genes (clone names M1, G2, 3M, 5G, 6G, 9G, 10G, 12G, 14G, and 15G) was observed in the second PCR (data not shown). Since the DNA of 3M and 5G were not amplified by PCR following Gibson assembly, in total, eight clones were evaluated, and each showed a higher signal with *E*. *coli* O26 than with other antigens in ELISA (Fig. 3b).

Using this Ecobody technology, we accomplished the whole process from collection of blood to ELISA within 2 days of normal daytime operations. From the results above, we selected anti-*V*. *parahaemolyticus* clone 22G, which appeared to be a superior mAb from the point of view of both high ELISA signal and low cross-reactivity, for further experiments.

### *E*. *coli in vivo* expression and refolding of Zipbody proteins

Next, to investigate whether the antibody obtained by Ecobody technology can be produced in an active form in an *E*. *coli in vivo* expression system, clone 22 G was expressed in *E*. *coli* strain SHuffle T7 Express. Although ELISA using the soluble fraction showed slight activity (data not shown), Coomassie Brilliant Blue (CBB) staining of the soluble and insoluble fractions after SDS-PAGE revealed that ≥ 90% of the produced Zipbody proteins appeared in the insoluble fraction (Supplementary Fig. S2a). Therefore, we decided to perform refolding of the insoluble fraction. The insoluble proteins were solubilised with 6 M guanidinium chloride (GuHCl) and refolding was carried out at a concentration of 1.6 mg/mL protein; about 83% was recovered in a soluble fraction. The protein band pattern on SDS-PAGE was almost the same as before refolding (Supplementary Fig. S2b). ELISA using the refolded product at various concentrations showed that it had high binding activity and specificity against the antigen *V*. *parahaemolyticus* (Supplementary Fig. S2c).

### Properties of refolded Zipbody proteins

**Figure 4:**
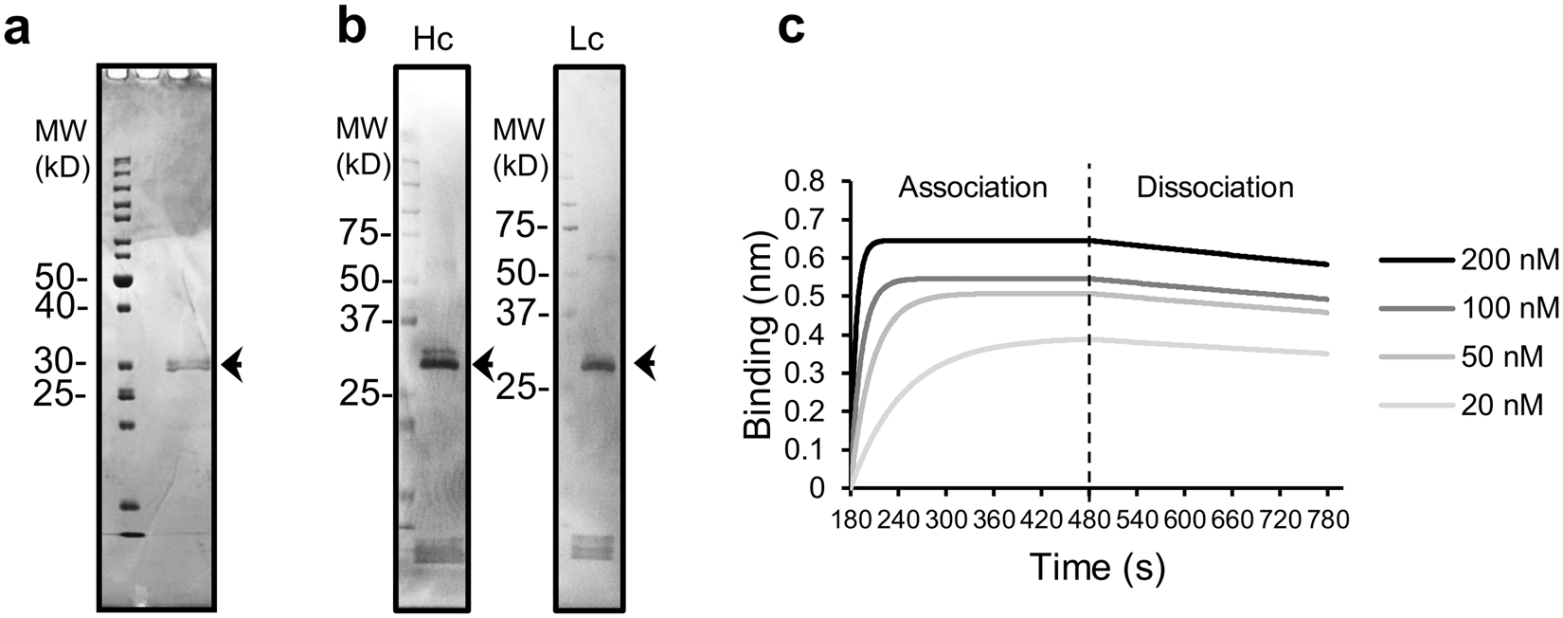
Analysis of refolded and purified Zipbody mAb produced in *E*. *coli*. Refolded and purified anti-*V*. *parahaemolyticus* Zipbody clone G22 produced in *E*. *coli* SHuffle T7 Express was used. The purified fraction in these analyses was obtained by Ni-affinity chromatography. The left-hand lane in panels a and b indicates molecular size markers. Arrows show the expected size of the Zipbody proteins (which overlap). (**a**) Coomassie Brilliant Blue staining after SDS-PAGE. One microgram of the purified fraction was loaded. (**b**) Western blotting detecting Hc and Lc, with anti-HA tag-HRP and anti-His tag-HRP conjugated antibodies, respectively. Loaded protein amount = 0.1 µg. (**c**) Blitz sensorgram at various Zipbody mAb concentrations against inactivated *V*. *parahaemolyticus* cells, corrected with 0 nM Zipbody as the reference.

In Zipbody, His and HA tags are attached at the C-termini of the Hc and Lc, respectively. Thus, the refolded product (1.3 mg of protein) was purified by Ni-affinity chromatography to obtain 0.42 mg of purified protein. Supplementary Table 1 summarises protein recovery during the purification steps. CBB staining after SDS-PAGE and western blotting detecting tags attached to the Hc and Lc showed clear bands at the expected sizes (Fig. 4a,b). These results indicated that the Zipbody proteins were purified as an association of Hc-Lc, and the chains almost overlapped on SDS-PAGE after separation. The apparent dissociation constant when inactivated *V*. *parahaemolyticus* cells were used as the antigen was calculated by the biolayer interferometry method^25^ and the respective values were: *K*_D_ = 469 pM, *k*_a_ = 7.34 × 10^5^ (1/Ms), *k*_d_ = 3.44 × 10^−4^ (1/s), *χ* ^2^ = 1.39, *R* ^2^ = 0.95. A sensorgram is shown in Fig. 4c. From these data, it was confirmed that the mAb obtained by Ecobody technology and produced by *E*. *coli* as a Zipbody had sufficient function as a mAb.

### DNA sequences of the mAbs

We confirmed the sequence diversity of each clone. DNA sequences of variable regions of the rabbit mAbs obtained in this study are available with accession nos. LC269739–LC269768.

## Discussion

Rapid screening of mAbs is important in accelerating research and industry. In this report, by employing two recent technologies, “Zipbody to promote association of Hc and Lc of Fab” and “Increase in protein expression by N-terminal SKIK peptide tag”, to overcome the low productivity of active Fab in the CFPS step in our SICREX method, we demonstrated that mAbs against a target antigen can be obtained from rabbits and evaluated within two days. We named this improved SICREX method “Ecobody technology”.

First, we found that an LZ fusion to Fab and attachment of an SKIK tag had a synergistic effect in producing active mAbs in CFPS (Fig. 2). In addition, DsbC was also effective in assisting the synthesis of active mAbs, as in previous cases of IgG and Fab production in CFPS^21,26^. These results suggest that: 1) The Hc and Lc are more likely to associate in the presence of the LZ; 2) the amount of synthesised protein is dramatically increased by the SKIK tag; and 3) formation of disulfide bonds by DsbC acted synergistically and activated Fab (Zipbody) synthesis. Thus, overall, ELISA evaluation became possible. When unmodified Fab is produced in CFPS, the formation efficiency is markedly different depending on the clone. In particular, rabbit antibodies are rich in Cys compared with antibodies from other animal species^18^, and it was difficult to synthesise active rabbit Fab in our previous study^17^. In some cases, the amount of Hc and Lc DNA templates should not be equivalent in CFPS, presumably because the expression levels of the two chains are different^21^. In fact, our preliminary study showed that, even if Hc and Lc alone were synthesised well, the synthesised amount of Hc became very low when both were coexpressed (data not shown). By attaching the SKIK tag, we could improve the coexpression level of both chains (Fig. 2b). Western blotting analysis of seven Zipbody proteins for anti-*V*. *parahaemolyticus* expressed in CFPS showed that they were well expressed almost equally except for Hc of clone 7 M (supplementary Fig. 3), and the estimated amount of protein produced in 10 μL scale CFPS was about 0.3 μg on average by calculation based on comparisons of band intensities on western blotting and ELISA signals of the purified proteins. This mAb yield (30 μg/mL) was better than that of animal cell HEK293 which showed that mAb concentrations greatly varied depending on the clones and the mean concentration was 617 ng/mL-culture^27^. They mentioned that it was enough for many further assays, including protein-based biochemical screens and cell-based functional assays. Therefore, the synthesized amount of mAbs in CFPS would be sufficient for just screening by ELISA in Ecobody technology. Our findings suggest that positive clones that may have been overlooked as having low activity due to insufficient protein synthesis may now be legitimately evaluated, and we believe that they are very important in mAb screening.

Using Ecobody technology, we demonstrated that it was possible to complete the process from isolation of B cells to mAb evaluation by manual operation in just two normal working days. The obtained mAb clones could be synthesised as Zipbody proteins with an SKIK tag in CFPS and had binding activity to relevant antigens (Fig. 3). Most of them had high specificity to the target antigens, probably because we removed B cells that cross-reacted with similar antigens in the cell selection step. This simple subtraction screening is an effective approach to obtain antigen-specific mAbs. While pathogenic bacteria was used as the antigen in this work, a rapid mAb screening by Ecobody technology will be available for challenging antigens like small chemicals, because B cells expressing mAbs against such antigens can be isolated by magnetic beads or FACS^8^. In addition, the most advantageous feature of Ecobody technology is that all the processes are carried out in tubes and only simple pipetting operations are required after B cell acquisition; therefore, the process can be easily be automated. This is a distinct advantage over other single B cell methods using animal cells, in which transfection and culturing of cells are necessary^4,8^. In this study, all processes were performed manually and single B cell selection using microscopy was laborious, but we expect that the screening system could become more efficient by introducing a single cell sorter and automatic pipetting machines.

One mAb clone (22 G) with good ELISA signals was selected and expression was performed in the *E*. *coli* strain SHuffle T7 Express. Most of the target proteins were produced in insoluble form, consistent with our previous study of the SKIK tag (Supplementary Fig. S2a)^24^. Therefore, we tried refolding the insoluble Zipbody by the method used for scFv^28^, and successfully recovered > 80% of the insoluble proteins in a soluble form without optimization of the refolding process (Supplementary Fig. S2b, Supplementary Table S1). His-tag purification of the refolded protein yielded about 8.5 mg of purified Zipbody from *E*. *coli* cells per litre of culture in LB medium. This refolding yield was comparable to some other reports^29,30^, although we cannot directly compare each case because the genes are different. Nonetheless, milligram-scale recovery of purified mAbs from 1 L culture is the highest level achieved in trials using *E*. *coli* expression systems^31^. The refolded and purified Zipbody had Hc-Lc association and the binding activity was sufficiently high (*K*_D_ = 469 pM) when compared with that of natural rabbit mAbs, which have *K*_D_ values of a few hundred pM (Fig. 4)^32^. The molecular structure and stability of the refolded mAbs, and the versatility of this method to obtain other mAb clones must be investigated, but we expect that rapid Ecobody technology coupled with *E*. *coli* expression has the potential to provide alternative Fab formats for practical use at low cost.

In conclusion, we adopted two techniques, Zipbody and the SKIK peptide tag, and succeeded in developing a mAb screening system, Ecobody technology, capable of generating and evaluating mAbs derived from one B cell very rapidly and efficiently. Although there are still issues to be improved, we believe that this rapid method can contribute to antibody research and industry.

## Methods

### Antigens

Bacteria *V*. *parahaemolyticus* NBRC 12711, *E*. *coli* O26 GTC14538 (verotoxin-1 producing strain), *E*. *coli* O157 GTC03904 (non-toxic), and *E*. *coli* O111 GTC14517 were used as antigens. They were purchased from the Biological Resource Center at the National Institute of Technology and Evaluation (NITE, Kisarazu, Japan) and the National BioResource Project GTC Collection (Gifu, Japan), cultured in Luria-Bertani (LB) medium at 37 °C overnight, and inactivated by incubation at 80 °C for 30 min in phosphate-buffered saline (PBS) containing 0.25% formalin. They were stored at −20 °C. Bacterial cells coated with magnetic beads for the B cell selection step were prepared as described previously^17^.

### Immunization of rabbits

New Zealand White rabbits (2–3 weeks) were immunised with 10^8^ inactivated bacterial cells supplemented with 2.5 mL of complete Freund’s adjuvant by hypodermic injection. The second and third boosters were given after intervals of 2 weeks and 10 days, respectively. Blood samples were collected 2 days after the final booster. This study was approved by the Committee of Animal Experiments of the Graduate School of Bioagricultural Sciences, Nagoya University (permit number 2015022611) and performed according to the Regulations on Animal Experiments in Nagoya University. For separating lymphocytes, we followed a previously reported protocol using density-gradient centrifugation^17^.

### Selection of B cells by FACS and antigen-coated magnetic beads

To select B cells producing mAbs, 10^6^ cells/mL-PBS were stained with 1 µM ER-Tracker™ Green (BODIPY^®^ FL Glibenclamide, Life Technologies, Foster City, CA) at room temperature for 5 min then centrifuged at 1,000 × *g* for 2 min as described previously^6^. The cells with higher fluorescence intensity were then sorted by FACS (JSAN; Bay Bioscience, Kobe, Japan). Next, the sorted cells were selected by antigen-coated magnetic beads as described previously^17^. In brief, biotinylated inactivated bacterial cells conjugated with DynaBeads M-280 Streptavidin (SA beads, Life Technologies) were prepared and these conjugates were used to concentrate B cells that bound to the antigens after removing cells non-specifically bound to SA-beads and *E*. *coli* DH5α-coated beads. After that, the remaining B cells were separated into each well (1 cell/10 µL-PBS) of 0.2 mm glass-bottomed 384-well imaging plates (Corning, Corning, NY, USA) and the bead-B cell complexes in each well were confirmed under a phase-contrast inverted microscope (CKX53; Olympus, Tokyo, Japan). Bürker-Türk plates were used for cell counting.

### Amplification of mAb genes from single B cells

Supplementary Table 2 lists all DNA primers used in this study. The following three steps were performed without a break:

i. Reverse transcription (RT). After removing the supernatant (9 µL) from each well of the 384-well glass plate, RT reaction mixture was added to each well containing single B cell-bead complexes. The reaction conditions were: 0.2 µM each gene specific primer, 0.5 mM each dNTP, 5 mM dithiothreitol, 2 U/µL RNaseOUT and 10 U/µL SuperScript IV (all purchased from Life Technologies) in a 10-µL mixture at 55 °C for 15 min.
ii. First PCR. Immediately after RT was finished, 0.5 µL of reaction mixture was used as the template for this PCR with gene specific primers for Lc and Hc, separately. Forward primers were designed to anneal to signal sequences. Reaction conditions were: 0.1 µM of each primer, 0.2 mM dNTPs, 0.05 U/µL LA *Taq* Hot Start version (Takara Bio, Otsu, Japan) in a Mg^2+^ supplemented buffer, in a 10-µL mixture. The thermal program was 94 °C (2 min); 30 cycles of 94 °C (30 s), 55 °C (30 s), and 72 °C (45 s); and finally 72 °C (2 min).
iii. Second PCR to connect tails required for the subsequent Gibson assembly step. One microlitre of the first PCR reaction mixtures were directly used as the templates. Reactions were carried out separately for Lc, Hc (IgG type), and Hc (IgM) as follows: 0.1 µM of each primer, Tks Gflex™ polymerase (Takara Bio) and the manufacturer’s recommended composition at 10 µL scale; thermal program: 94 °C (2 min); 30 cycles of 98 °C (10 s), 60 °C (15 s), and 68 °C (20 s); and finally 68 °C (2 min).

### Construction of DNA fragments for CFPS

Continuously, from the previous DNA amplification steps, we prepared DNA fragments for CFPS and plasmids for transformation followed by sequence analysis.

iv. Gibson assembly of the second PCR product and linearised pRSETb vector containing (i) the SKIK tag immediately after the start codon, (ii) fragments encoding Lc or Hc (IgM or IgG) fused with the leucine zipper A (LZA) for Hc and B (LZB) for Lc, and (iii) HA or His tags downstream of the LZ DNA (Fig. 1). The Gibson assembly reagent was purchased from New England Biolabs (Ipswich, MA). After mixing 1 µL of the second PCR reaction mixture, 1 µL of the linearised vector (100 ng), and 2 µL of 2 × Gibson Master Mix, they were incubated at 50 °C for 15 min. The genes in the vector were artificially synthesised for optimised codon usage for *E*. *coli* expression and embedded into pRSETb (Life Technologies, DNA sequences of linearized vectors are available in Supplementary Information). The linearised DNA fragments used here were amplified using Pyrobest™ Polymerase (Takara Bio). Purified DNA fragments were stored at −20 °C until use.
v. Preparation of DNA fragments for CFPS. DNA fragments containing the T7 promoter, gene (Lc or Hc), and T7 terminator were then amplified from the assembled products by PCR with Tsk Gflex polymerase. The amplified DNA fragments were directly used as the templates for the following CFPS step.

### Plasmid preparation and DNA sequence analysis

To analyse DNA sequences of the obtained mAbs, *E*. *coli* XL10-Gold (Agilent Technologies, Santa Clara, CA, USA) was transformed with 1 µL of the Gibson assembled constructs (circular expression plasmids) for plasmid preparation. Colonies were selected on LB-medium plates containing 100 µg/mL ampicillin. The same primers as for construction of the CFPS template were used for sequencing.

### Cell-free protein synthesis

The reconstituted *E*. *coli*-based CFPS PURE system (GeneFrontier, Kashiwa, Japan), with or without DsbC, was used. Oxidised glutathione (GSSG) was also included when DsbC was added to the reaction mixture, as recommended by the manufacturer. Proteins were expressed on a 10-µL scale containing 0.5 µL PCR product or 20 ng purified DNA fragments (each of Hc and Lc) as the template, at 37 °C for 90 min, using a thermal cycler. FluoroTect™ GreenLys *in vitro* Translation Labeling System (Promega, Madison, WI) was included to confirm protein synthesis in subsequent SDS-PAGE analysis, as described previously^17^.

### SDS-PAGE and ELISA

Unless otherwise stated, mAbs produced in CFPS or *E*. *coli* were analysed by SDS-PAGE in reducing conditions and ELISA using our standard protocol described in reference^24^. In brief, proteins separated on SDS-PAGE were visualised by CBB staining, western blotting detecting tags using horseradish peroxidase (HRP)-conjugated antibodies (anti-FLAG tag-HRP [GeneTex, Irvine, CA], anti-His tag-HRP [Medical and Biological Laboratories, Nagoya, Japan], or anti-HA tag-HRP [Wako, Osaka, Japan]) and 1-Step™ TMB-Blotting Substrate Solution (Thermo, Waltham, MA), or fluorescent imaging. In ELISA, 50 µL of inactivated bacterial cells (OD_600 nm_ = 0.1) or 0.4% BSA in PBS were coated on the MaxiSorp plate (Thermo) overnight, and we followed the standard ELISA protocol using Zipbody as the primary antibody, and anti-rabbit Ig(G + M)-HRP conjugate (Southern Biotech, Birmingham, AL) or anti-His tag-HRP conjugate as the secondary antibody. Can Get Signal Immunoreaction Enhancer Solution 2 (Toyobo, Osaka, Japan) was used for dilution of the secondary antibody.

### Production of Zipbody proteins in *E*. *coli*

The plasmid for coexpression of Hc and Lc in a pET-based system was constructed as follows: Hc and Lc genes were respectively amplified from the pRSET-based plasmids constructed in step 10 of Fig. 1, followed by three-DNA-fragment Gibson assembly with linearised pET22b vector. The expression of Zipbody with an SKIK tag was carried out using *E*. *coli* SHuffle T7 Express as the host, grown in 50 mL LB medium containing 100 μg/mL ampicillin with 1 mM IPTG induction at 16 °C for 24 h, as described in our previous study^24^.

### Refolding and purification of Zipbody

The above expression cells were collected by centrifugation and completely solubilised in solubilization buffer (6 M GuHCl, 100 mM Tris-HCl [pH 8.0], 200 mM NaCl, and 10 mM 2-mercaptoethanol) with sonication at room temperature. Then we essentially followed the previously reported dialysis refolding method for scFv by stepwise decrease of the GuHCl concentration^28^, except l-Arg was not used. In brief, 0.95 mL of solubilised protein solution was dialyzed against 100 mL of outer solution (in successive steps 6 M, 3 M, 2 M, 1 M, 0.5 M and 0 M GuHCl, in 100 mM Tris-HCl [pH 8.0] and 200 mM NaCl) each for 12 h at 4 °C. At the 1 M GuHCl stage, 375 µM GSSG was included in the outer solution. The solubilised fraction after finishing all processes was separated by centrifugation at 15,000 × *g* for 10 min. The refolded Zipbody was purified with a Ni-Sepharose 6 Fast Flow resin (GE healthcare, Little Chalfont, UK)-embedded column (resin volume 1 mL), using binding buffer (20 mM imidazole, 0.5 M NaCl, 20 mM Na-phosphate buffer [pH 7.4]) and elution buffer (0.5 M imidazole in the same buffer), according to the manufacturer’s recommended protocol. In all steps, protein concentration was determined by Bradford assay with BSA as the standard.

### Kinetic analysis of Zipbody produced in *E*. *coli*

Kinetic analysis was performed based on biolayer interferometry by using BLItz (Pall ForteBio, Menlo Park, CA) at room temperature with an aminopropylsilane sensor, as follows: (i) the sensor was washed with 400 µL of PBS for 30 s; (ii) inactivated *V*. *parahaemolyticus* cells in PBS (OD_600 nm_ = 1.0) were immobilized on the sensor for 120 s; (iii) the sensor was washed with 400 µL PBS for 30 s; (iv) purified Zipbody (4 µL) at various concentrations (0, 20, 50, 100, and 500 nM) diluted with 1 × kinetics buffer (Pall ForteBio) was associated with the sensor for 300 s; (v) Zipbody was then dissociated in 400 µL PBS for 300 s. The *k*_a_, *k*_d_, and *K*_D_ values were determined by a global fitting mode in a 1:1 binding model.

### Availability of data

All data generated or analysed in this study are included in this published article and its Supplementary Information. The datasets are also available from the corresponding authors on reasonable request.

## Electronic supplementary material

Supplementary information
