# Supplementary material for: Ecobody technology: rapid monoclonal antibody screening method from single B cells using cell-free protein synthesis for antigen-binding fragment formation
Source: Sci Rep. 2017 Oct 25;7:13979. doi: 10.1038/s41598-017-14277-0 (PMC5656612; doi:10.1038/s41598-017-14277-0)
Supplement: Supplementary file 1 — Supplementary information [file 41598_2017_14277_MOESM1_ESM.pdf]

Supplementary Information

Ecobody technology: rapid monoclonal antibody screening method from single B cells using cell-free protein synthesis for antigen-binding fragment formation

Teruyo Ojima-Kato[1a*], Satomi Nagai[1], Hideo Nakano[1b*]

[1]*Graduate School of Bioagricultural Sciences, Nagoya University, Furo-cho, Chikusa-ku, Nagoya 464-8601, Japan*

[*] Correspondence should be addressed to

[a] Email: teruyo.ojima@gmail.com, Tel: +81-52-789-4144, Fax: +81-52-789-4145 or

[b] Email: hnakano@agr.nagoya-u.ac.jp, Tel: +81-52-789-4144, Fax: +81-52-789-4145

Supplementary Table S1. Recovery ratio of *E. coli* expressed proteins.

Step	Protein concentration (mg/mL)	Total volume (mL)	Amount of protein (mg)	Recovery ratio (%)
Solubilization by 6 M GuHCl	1.62	0.95	1.54	
Refolding	0.98	1.3	1.28	82.8
His tag purification	0.094	4.5	0.423	27.4

Inclusion bodies produced in *E. coli* SHuffle T7 Express in 50 mL of LB medium supplemented with ampicillin were used. The solubilized amount of protein is regarded as 100% in recovery ratio.

Supplementary Table 2. Primers used in this study.

Description	Target gene	Sequence (5'-3')
Reverse transcription	Lc	ACAGTCACCCCTATTGAAGCT
		GCAGTCACCCCTGTTGAAGCT
		ACAGTCTCCCCTATTGAAGCT
	Hc (IgG)	CCATGTGAACTGCACCTCGG
	Hc (IgM)	GGGAAAGGACACTCGCA
First PCR	Lc	ATGGACACGAGGGCCCCCAC
		ATGGACATGAGGGCCCCCAC
		ATGGACACGAGGGCACCCGC
		GTGCCCTGGGTCACCTTGCAG
		GTGTCCTGGGTCACCTTGCAGGT
		CCCTCGTGCGTGACCTGGCA
	Hc (both IgG and IgM)	ATGGAGACTGGGCTGCGCTGG
	Hc (IgG)	TGACCTCGGGGGTGCGTGAGAT
	Hc (IgM)	GGTACTCCTCTGTGCCCTGGAGGA
Second PCR	Lc	CATATGTCTAAAATAAAAGCSCTTGTGATGACCCAGACTC
		CATATGTCTAAAATAAAAGCAGCCGTGCTGCTGACCCAGA
		CATATGTCTAAAATAAAAGACCCTRTGCTGACCCAGACTC
		CATATGTCTAAAATAAAAGCTCAAGTGCTGACCCAGACTC
		CATATGTCTAAAATAAAAGCTGACATTGTGATGACCCAGA
		CATATGTCTAAAATAAAAGCATTCGAATTGACCCAGACTC
		CATATGTCTAAAATAAAAGATGTTGTGATGACCCAGACTC
		CATATGTCTAAAATAAAAGCCTATGATATGACCCAGACTC
		CATATGTCTAAAATAAAAGCCATCGAAATGACCCAGACTC
		CATATGTCTAAAATAAAAGCAGCCGTGCTGACCCAGACAC
		CATATGTCTAAAATAAAAGCCCAAGTGCTGACCCAGACTC
		CATATGTCTAAAATAAAAGCCGAAGTAGTGATGACCCAGA
		CATATGTCTAAAATAAAAGCCAACATCGTGCTGACCCGTA
		CATATGTCTAAAATAAAAGGTGCCATCCAGATGACCCAGT
		AGTATTTATTGGCAACACACACGATGGTGRCTGTTYCAGTTG
	Hc (both IgG and IgM)	CATATGTCTAAAATAAAACAGCAGCTGGAGCAGTCCG
		CATATGTCTAAAATAAAACAGTCGGTGAAGGAGTCCG
		CATATGTCTAAAATAAAACAGTCGTTGGAGGAGTCCG

		CATATGTCTAAAATAAAACAGTCGCTGGAGGAGTCCG
		CATATGTCTAAAATAAAACAGGAGCAGCTGAAGGAG
		CATATGTCTAAAATAAAACAGTCGGTGAAGGACTCCG
		CATATGTCTAAAATAAAACAGGAGCAGTTGAAGGAGTC
		CATATGTCTAAAATAAAACAGTCGGTGGAGGAGTCC
		CATATGTCTAAAATAAAACAGTCCTTGGAGGAGTCC
		CATATGTCTAAAATAAAACAGGAGCAGCTGGAGGAG
		CATATGTCTAAAATAAAACAGCCGTTGGAGGAGTCC
		CATATGTCTAAAATAAAACAGTCGTCGGAGGAGTCC
		CATATGTCTAAAATAAAACAGGCGTTGGAGGAGTCC
		CATATGTCTAAAATAAAACAGCAGCAGCTGATGGAG
		CATATGTCTAAAATAAAACAGCAGCAGCTGGTGGAG
		CATATGTCTAAAATAAAACAGGAGCAGCTGGTGCAG
	Hc (IgG)	ACCACAACACGGTGCCAGTGGGAAGACTGATGGAGCCTT
		ACCACAACACGGTGCCAGTGGGAAGACTGACGGAGCCTT
	Hc (IgM)	GCTAACCAGCGGATACAGAGTTGGAGATGACAGGCTCAC
Amplification of pRSET-based vector fragment for Gibson assembly	Lc	TGTGTTGCCAATAAATACT
		TTTTATTTTAGACATATGTATATCTCCTTCTTAAAGTTAAACAAAAT
	Hc (IgG)	CTGGCACCGTGTTGTGGTG
		TTTTATTTTAGACATATGTATATCTCCTTCTTAAAGTTAAACAAAAT
	Hc (IgM)	CTGTATCCGCTGGTTAGCTGT
		TTTTATTTTAGACATATGTATATCTCCTTCTTAAAGTTAAACAAAAT
Construction of expression plasmid of clone 22G	Hc	GATCCGGCTGCTAACAAAGCCC
		ATGATATCTCCTTCTAGATTATTAAGCGTAATCTGGAAC
	Lc	TCTAGAAGGAGATATCATATGTCTAAAATAAAAGATGTTG
		GGGCTTTGTTAGCAGCCGGATC
	pET22b-vector	GATCCGGCTGCTAACAAAGCCC
		TTTTATTTTAGACATATGTATATCTCCTTCTTAAAG
Amplification of templates for CFPS, F1		ATCTCGATCCCGCGAAATTAATACG
R1		TCCGGATATAGTTCCTCCTTTCAG

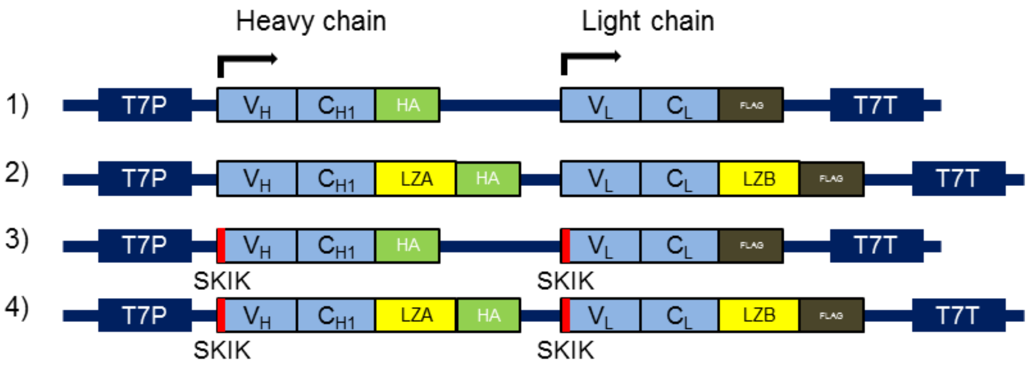

Supplementary Fig. S1. DNA templates used for CFPS.

Four variations of rabbit anti-*Listeria monocytogenes* monoclonal antibody genes used to assess the effect of leucine zipper fusion and an N-terminal SKIK peptide tag in CFPS. Arrows indicate start positions for open reading frames of heavy and light chains.T7P: T7 promoter, HA: HA tag, FLAG: FLAG tag, T7T: T7 terminator, LZA: leucine zipper A, LZB: leucine zipper B, SKIK: SKIK tag.

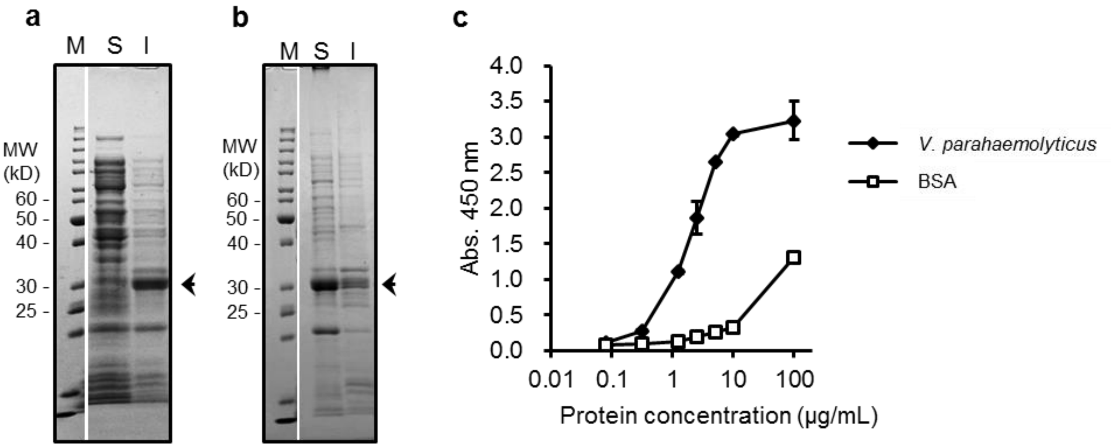

Supplementary Fig. S2. Analyses of the *E. coli*-expressed and refolded proteins.

Anti- *V. parahaemolyticus* clone 22G was expressed in *E. coli* as Zipbody with the SKIK tag. M, size markers; Arrow, expected size of overlapping Hc and Lc bands of Zipbody mAb.

a) CBB staining of the soluble (S) and insoluble (I) fraction expressed in *E. coli* SHuffle T7 Express on SDS-PAGE.

b) CBB staining of the soluble (S) and insoluble (I) fractions after refolding.

c) ELISA results for the refolded Zipbody mAb. The solubilized fraction after refolding was analysed by ELISA at various concentrations with *V. parahaemolyticus* and BSA as the antigens.

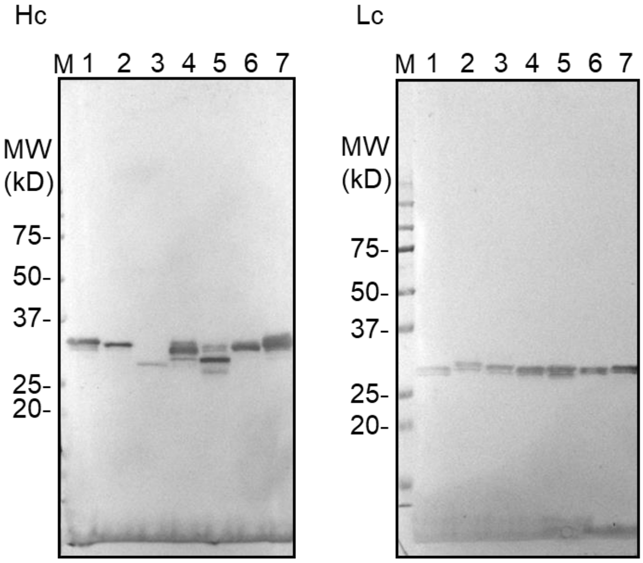

Supplementary Fig. S3. Full western blotting images of 1 μL of anti-*V. parahaemolyticus* Zipbody proteins produced in CFPS.

Hc and Lc were visualised with anti-HA tag-HRP and anti-His tag-HRP conjugated antibodies, respectively. Sample numbers 1-7 correspond to clones 3G, 5M, 7M, 20G, 22G, 30M, and 36G. The left-hand lane in each image indicates molecular size markers.

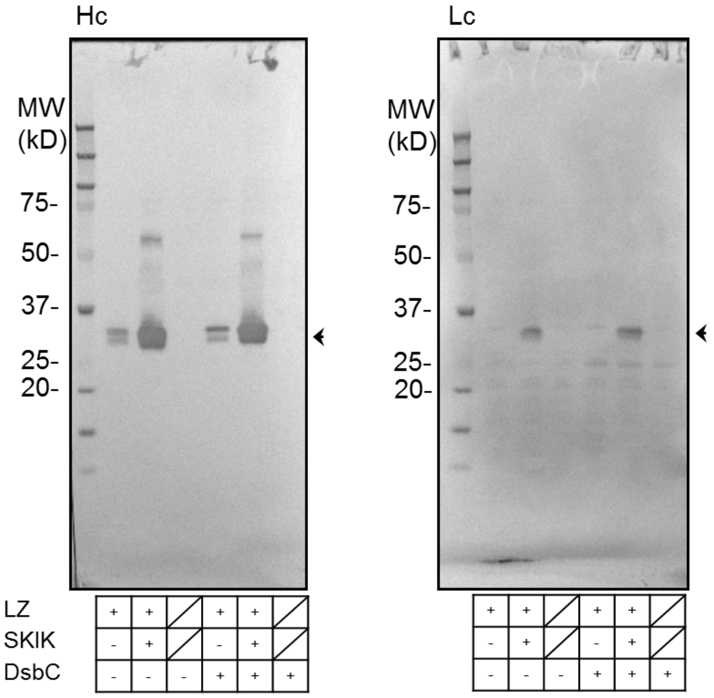

Supplementary Fig. S4. Full western blotting images of Figure 2b.

Arrows indicate the target antibody fragments used for Image J analysis. The left-hand lane in each image indicates molecular size markers.

Supplementary Information for DNA sequences.

End points in the linearised vector are indicated with red slant lines.

```
>pRSET-IgGCH1-LZA-HAtag
GATCTCGATCCCGCGAAATTAATACGACTCACTATAGGGAGACCACAACGGTTTCCCTCTAGAAATAATTTTGTTTAACTTTAAGAAG
GAGATATACATATGTCTAAAATAAAA/CTGGCACCGTGTTGTGGTGATACCCCGAGCAGCACCGTTACCCTGGGTTGTCTGGTTAAAG
GTTATCTGCCGGAACCGGTTACCGTTACCTGGAATAGCGGCACCCTGACCAATGGTGTTCGTACCTTTCCGAGCGTTCGTCAGAGCAG
CGGTCTGTATAGCCTGAGCAGCGTTGTTAGCGTTACCAGCAGCAGCCAGCCGGTTACCTGTAATGTTGCACATCCGGCAACCAATACC
AAAGTTGATAAAACCGTTGCACCGAGCACCTGTGGCGGTGGTGGGAGCGCCCAGCTCGAAAAGGAGCTGCAAGCCCTGGAGAAGGAGA
ACGCCCAGCTCGAATGGGAGCTCCAGGCCCTGGAGAAGGAGCTGGCCCAGAAGGGCGGTACCATGTACCCATACGATGTTCCAGATTA
CGCTTAATAATCTAGAACTTGATCCGGCTGCTAACAAAGCCCGAAAGGAAGCTGAGTTGGCTGCTGCCACCGCTGAGCAATAACTAGC
ATAACCCCTTGGGGCCTCTAAACGGGTCTTGAGGGGTTTTTTGCTGAAAGGAGGAACTATATCCGGATCTGGCGTAATAGCGAAGAGG
CCCGCACCGATCGCCCTTCCCAACAGTTGCGCAGCCTGAATGGCGAATGGGACGCGCCCTGTAGCGGCGCATTAAGCGCGGCGGGTGT
GGTGGTTACGCGCAGCGTGACCGCTACACTTGCCAGCGCCCTAGCGCCCGCTCCTTTCGCTTTCTTCCCTTCCTTTCTCGCCACGTTC
GCCGGCTTTCCCCGTCAAGCTCTAAATCGGGGGCTCCCTTTAGGGTTCCGATTTAGTGCTTTACGGCACCTCGACCCCAAAAAACTTG
ATTAGGGTGATGGTTCACGTAGTGGGCCATCGCCCTGATAGACGGTTTTTCGCCCTTTGACGTTGGAGTCCACGTTCTTTAATAGTGG
ACTCTTGTTCCAAACTGGAACAACACTCAACCCTATCTCGGTCTATTCTTTTGATTTATAAGGGATTTTGCCGATTTCGGCCTATTGG
TTAAAAAATGAGCTGATTTAACAAAAATTTAACGCGAATTTTAACAAAATATTAACGCTTACAATTTAGGTGGCACTTTTCGGGGAAA
TGTGCGCGGAACCCCTATTTGTTTATTTTTCTAAATACATTCAAATATGTATCCGCTCATGAGACAATAACCCTGATAAATGCTTCAA
TAATATTGAAAAAGGAAGAGTATGAGTATTCAACATTTCCGTGTCGCCCTTATTCCCTTTTTTGCGGCATTTTGCCTTCCTGTTTTTG
CTCACCCAGAAACGCTGGTGAAAGTAAAAGATGCTGAAGATCAGTTGGGTGCACGAGTGGGTTACATCGAACTGGATCTCAACAGCGG
TAAGATCCTTGAGAGTTTTCGCCCCGAAGAACGTTTTCCAATGATGAGCACTTTTAAAGTTCTGCTATGTGGCGCGGTATTATCCCGT
ATTGACGCCGGGCAAGAGCAACTCGGTCGCCGCATACACTATTCTCAGAATGACTTGGTTGAGTACTCACCAGTCACAGAAAAGCATC
TTACGGATGGCATGACAGTAAGAGAATTATGCAGTGCTGCCATAACCATGAGTGATAACACTGCGGCCAACTTACTTCTGACAACGAT
CGGAGGACCGAAGGAGCTAACCGCTTTTTTGCACAACATGGGGGATCATGTAACTCGCCTTGATCGTTGGGAACCGGAGCTGAATGAA
GCCATACCAAACGACGAGCGTGACACCACGATGCCTGTAGCAATGGCAACAACGTTGCGCAAACTATTAACTGGCGAACTACTTACTC
TAGCTTCCCGGCAACAATTAATAGACTGGATGGAGGCGGATAAAGTTGCAGGACCACTTCTGCGCTCGGCCCTTCCGGCTGGCTGGTT
TATTGCTGATAAATCTGGAGCCGGTGAGCGTGGGTCTCGCGGTATCATTGCAGCACTGGGGCCAGATGGTAAGCCCTCCCGTATCGTA
GTTATCTACACGACGGGGAGTCAGGCAACTATGGATGAACGAAATAGACAGATCGCTGAGATAGGTGCCTCACTGATTAAGCATTGGT
AACTGTCAGACCAAGTTTACTCATATATACTTTAGATTGATTTAAAACTTCATTTTTAATTTAAAAGGATCTAGGTGAAGATCCTTTT
TGATAATCTCATGACCAAAATCCCTTAACGTGAGTTTTCGTTCCACTGAGCGTCAGACCCCGTAGAAAAGATCAAAGGATCTTCTTGA
GATCCTTTTTTTCTGCGCGTAATCTGCTGCTTGCAAACAAAAAAACCACCGCTACCAGCGGTGGTTTGTTTGCCGGATCAAGAGCTAC
CAACTCTTTTTCCGAAGGTAACTGGCTTCAGCAGAGCGCAGATACCAAATACTGTTCTTCTAGTGTAGCCGTAGTTAGGCCACCACTT
CAAGAACTCTGTAGCACCGCCTACATACCTCGCTCTGCTAATCCTGTTACCAGTGGCTGCTGCCAGTGGCGATAAGTCGTGTCTTACC
GGGTTGGACTCAAGACGATAGTTACCGGATAAGGCGCAGCGGTCGGGCTGAACGGGGGGTTCGTGCACACAGCCCAGCTTGGAGCGAA
CGACCTACACCGAACTGAGATACCTACAGCGTGAGCTATGAGAAAGCGCCACGCTTCCCGAAGGGAGAAAGGCGGACAGGTATCCGGT
AAGCGGCAGGGTCGGAACAGGAGAGCGCACGAGGGAGCTTCCAGGGGGAAACGCCTGGTATCTTTATAGTCCTGTCGGGTTTCGCCAC
CTCTGACTTGAGCGTCGATTTTTGTGATGCTCGTCAGGGGGGCGGAGCCTATGGAAAAACGCCAGCAACGCGGCCTTTTTACGGTTCC
TGGCCTTTTGCTGGCCTTTTGCTCACATGTTCTTTCCTGCGTTATCCCCTGATTCTGTGGATAACCGTATTACCGCCTTTGAGTGAGC
TGATACCGCTCGCCGCAGCCGAACGACCGAGCGCAGCGAGTCAGTGAGCGAGGAAGCGGAAGAGCGCCCAATACGCAAACCGCCTCTC
CCCGCGCGTTGGCCGATTCATTAATGCAG
```

```
>pRSET-IgMCH1-LZA-HAtag
GATCTCGATCCCGCGAAATTAATACGACTCACTATAGGGAGACCACAACGGTTTCCCTCTAGAAATAATTTTGTTTAACTTTAAGAAG
GAGATATACATATGTCTAAAATAAAA/CTGTATCCGCTGGTTAGCTGTGAAGGTGCACTGACCGATGGTAATCTGGTTGCAATGGGTT
GTCTGGCACGTGATTTTCTGCCGAGCAGCGTTACCTTTAGCTGGTCCTTTAAAAACAACAGCGAAATTAGCAGCCGTACCGTTCGTAC
CTTTCCGGTTGTTAAACGTGGTGATAAATACATGGCAACCAGCCAGGTTCTGGTTCCGAGCAAAGATGTTCTGCAGGGCACCGAAGAA
TATCTGGTTTGTAAAGTTCAGCATAGCAATAGCAATCGTGATCTGCGTGTTAGCTTTCCGGGCGGTGGTGGGAGCGCCCAGCTCGAAA
AGGAGCTGCAAGCCCTGGAGAAGGAGAACGCCCAGCTCGAATGGGAGCTCCAGGCCCTGGAGAAGGAGCTGGCCCAGAAGGGCGGTAC
CATGTACCCATACGATGTTCCAGATTACGCTTAATAATCTAGAACTTGATCCGGCTGCTAACAAAGCCCGAAAGGAAGCTGAGTTGGC
TGCTGCCACCGCTGAGCAATAACTAGCATAACCCCTTGGGGCCTCTAAACGGGTCTTGAGGGGTTTTTTGCTGAAAGGAGGAACTATA
TCCGGATCTGGCGTAATAGCGAAGAGGCCCGCACCGATCGCCCTTCCCAACAGTTGCGCAGCCTGAATGGCGAATGGGACGCGCCCTG
TAGCGGCGCATTAAGCGCGGCGGGTGTGGTGGTTACGCGCAGCGTGACCGCTACACTTGCCAGCGCCCTAGCGCCCGCTCCTTTCGCT
TTCTTCCCTTCCTTTCTCGCCACGTTCGCCGGCTTTCCCCGTCAAGCTCTAAATCGGGGGCTCCCTTTAGGGTTCCGATTTAGTGCTT
TACGGCACCTCGACCCCAAAAAACTTGATTAGGGTGATGGTTCACGTAGTGGGCCATCGCCCTGATAGACGGTTTTTCGCCCTTTGAC
GTTGGAGTCCACGTTCTTTAATAGTGGACTCTTGTTCCAAACTGGAACAACACTCAACCCTATCTCGGTCTATTCTTTTGATTTATAA
GGGATTTTGCCGATTTCGGCCTATTGGTTAAAAAATGAGCTGATTTAACAAAAATTTAACGCGAATTTTAACAAAATATTAACGCTTA
CAATTTAGGTGGCACTTTTCGGGGAAATGTGCGCGGAACCCCTATTTGTTTATTTTTCTAAATACATTCAAATATGTATCCGCTCATG
AGACAATAACCCTGATAAATGCTTCAATAATATTGAAAAAGGAAGAGTATGAGTATTCAACATTTCCGTGTCGCCCTTATTCCCTTTT
TTGCGGCATTTTGCCTTCCTGTTTTTGCTCACCCAGAAACGCTGGTGAAAGTAAAAGATGCTGAAGATCAGTTGGGTGCACGAGTGGG
TTACATCGAACTGGATCTCAACAGCGGTAAGATCCTTGAGAGTTTTCGCCCCGAAGAACGTTTTCCAATGATGAGCACTTTTAAAGTT
CTGCTATGTGGCGCGGTATTATCCCGTATTGACGCCGGGCAAGAGCAACTCGGTCGCCGCATACACTATTCTCAGAATGACTTGGTTG
AGTACTCACCAGTCACAGAAAAGCATCTTACGGATGGCATGACAGTAAGAGAATTATGCAGTGCTGCCATAACCATGAGTGATAACAC
TGCGGCCAACTTACTTCTGACAACGATCGGAGGACCGAAGGAGCTAACCGCTTTTTTGCACAACATGGGGGATCATGTAACTCGCCTT
GATCGTTGGGAACCGGAGCTGAATGAAGCCATACCAAACGACGAGCGTGACACCACGATGCCTGTAGCAATGGCAACAACGTTGCGCA
AACTATTAACTGGCGAACTACTTACTCTAGCTTCCCGGCAACAATTAATAGACTGGATGGAGGCGGATAAAGTTGCAGGACCACTTCT
GCGCTCGGCCCTTCCGGCTGGCTGGTTTATTGCTGATAAATCTGGAGCCGGTGAGCGTGGGTCTCGCGGTATCATTGCAGCACTGGGG
CCAGATGGTAAGCCCTCCCGTATCGTAGTTATCTACACGACGGGGAGTCAGGCAACTATGGATGAACGAAATAGACAGATCGCTGAGA
TAGGTGCCTCACTGATTAAGCATTGGTAACTGTCAGACCAAGTTTACTCATATATACTTTAGATTGATTTAAAACTTCATTTTTAATT
TAAAAGGATCTAGGTGAAGATCCTTTTTGATAATCTCATGACCAAAATCCCTTAACGTGAGTTTTCGTTCCACTGAGCGTCAGACCCC
GTAGAAAAGATCAAAGGATCTTCTTGAGATCCTTTTTTTCTGCGCGTAATCTGCTGCTTGCAAACAAAAAAACCACCGCTACCAGCGG
TGGTTTGTTTGCCGGATCAAGAGCTACCAACTCTTTTTCCGAAGGTAACTGGCTTCAGCAGAGCGCAGATACCAAATACTGTTCTTCT
AGTGTAGCCGTAGTTAGGCCACCACTTCAAGAACTCTGTAGCACCGCCTACATACCTCGCTCTGCTAATCCTGTTACCAGTGGCTGCT
GCCAGTGGCGATAAGTCGTGTCTTACCGGGTTGGACTCAAGACGATAGTTACCGGATAAGGCGCAGCGGTCGGGCTGAACGGGGGGTT
CGTGCACACAGCCCAGCTTGGAGCGAACGACCTACACCGAACTGAGATACCTACAGCGTGAGCTATGAGAAAGCGCCACGCTTCCCGA
AGGGAGAAAGGCGGACAGGTATCCGGTAAGCGGCAGGGTCGGAACAGGAGAGCGCACGAGGGAGCTTCCAGGGGGAAACGCCTGGTAT
CTTTATAGTCCTGTCGGGTTTCGCCACCTCTGACTTGAGCGTCGATTTTTGTGATGCTCGTCAGGGGGGCGGAGCCTATGGAAAAACG
CCAGCAACGCGGCCTTTTTACGGTTCCTGGCCTTTTGCTGGCCTTTTGCTCACATGTTCTTTCCTGCGTTATCCCCTGATTCTGTGGA
TAACCGTATTACCGCCTTTGAGTGAGCTGATACCGCTCGCCGCAGCCGAACGACCGAGCGCAGCGAGTCAGTGAGCGAGGAAGCGGAA
GAGCGCCCAATACGCAAACCGCCTCTCCCCGCGCGTTGGCCGATTCATTAATGCAG
```

```
>pRSET-CL-LZB-Histag
GATCTCGATCCCGCGAAATTAATACGACTCACTATAGGGAGACCACAACGGTTTCCCTCTAGAAATAATTTTGTTTAACTTTAAGAAG
GAGATATACATATGTCTAAAATAAAA/TGTGTTGCCAATAAATACTTTCCGGATGTTACCGTTACCTGGGAAGTTGATGGCACCACCC
AGACCACCGGTATTGAAAATAGCAAAACACCGCAGAATAGCGCAGATTGTACCTATAATCTGAGCAGCACCCTGACCCTGACCAGCAC
CCAGTATAACAGCCATAAAGAATATACCTGCAAAGTGACCCAGGGTACAACCAGCGTTGTTCAGAGCTTTAATCGTGGTGATTGTGGC
GGTGGTGGGAGCGCCCAGCTCAAGAAGAAGCTGCAAGCCCTGAAGAAGAAGAACGCCCAGCTCAAGTGGAAGCTCCAGGCCCTGAAGA
AGAAGCTGGCCCAGAAGGGCGGTTCCCATCATCATCACCATCACTAATAATCGACTTGATCCGGCTGCTAACAAAGCCCGAAAGGAAG
CTGAGTTGGCTGCTGCCACCGCTGAGCAATAACTAGCATAACCCCTTGGGGCCTCTAAACGGGTCTTGAGGGGTTTTTTGCTGAAAGG
AGGAACTATATCCGGATCTGGCGTAATAGCGAAGAGGCCCGCACCGATCGCCCTTCCCAACAGTTGCGCAGCCTGAATGGCGAATGGG
ACGCGCCCTGTAGCGGCGCATTAAGCGCGGCGGGTGTGGTGGTTACGCGCAGCGTGACCGCTACACTTGCCAGCGCCCTAGCGCCCGC
TCCTTTCGCTTTCTTCCCTTCCTTTCTCGCCACGTTCGCCGGCTTTCCCCGTCAAGCTCTAAATCGGGGGCTCCCTTTAGGGTTCCGA
TTTAGTGCTTTACGGCACCTCGACCCCAAAAAACTTGATTAGGGTGATGGTTCACGTAGTGGGCCATCGCCCTGATAGACGGTTTTTC
GCCCTTTGACGTTGGAGTCCACGTTCTTTAATAGTGGACTCTTGTTCCAAACTGGAACAACACTCAACCCTATCTCGGTCTATTCTTT
TGATTTATAAGGGATTTTGCCGATTTCGGCCTATTGGTTAAAAAATGAGCTGATTTAACAAAAATTTAACGCGAATTTTAACAAAATA
TTAACGCTTACAATTTAGGTGGCACTTTTCGGGGAAATGTGCGCGGAACCCCTATTTGTTTATTTTTCTAAATACATTCAAATATGTA
TCCGCTCATGAGACAATAACCCTGATAAATGCTTCAATAATATTGAAAAAGGAAGAGTATGAGTATTCAACATTTCCGTGTCGCCCTT
ATTCCCTTTTTTGCGGCATTTTGCCTTCCTGTTTTTGCTCACCCAGAAACGCTGGTGAAAGTAAAAGATGCTGAAGATCAGTTGGGTG
CACGAGTGGGTTACATCGAACTGGATCTCAACAGCGGTAAGATCCTTGAGAGTTTTCGCCCCGAAGAACGTTTTCCAATGATGAGCAC
TTTTAAAGTTCTGCTATGTGGCGCGGTATTATCCCGTATTGACGCCGGGCAAGAGCAACTCGGTCGCCGCATACACTATTCTCAGAAT
GACTTGGTTGAGTACTCACCAGTCACAGAAAAGCATCTTACGGATGGCATGACAGTAAGAGAATTATGCAGTGCTGCCATAACCATGA
GTGATAACACTGCGGCCAACTTACTTCTGACAACGATCGGAGGACCGAAGGAGCTAACCGCTTTTTTGCACAACATGGGGGATCATGT
AACTCGCCTTGATCGTTGGGAACCGGAGCTGAATGAAGCCATACCAAACGACGAGCGTGACACCACGATGCCTGTAGCAATGGCAACA
ACGTTGCGCAAACTATTAACTGGCGAACTACTTACTCTAGCTTCCCGGCAACAATTAATAGACTGGATGGAGGCGGATAAAGTTGCAG
GACCACTTCTGCGCTCGGCCCTTCCGGCTGGCTGGTTTATTGCTGATAAATCTGGAGCCGGTGAGCGTGGGTCTCGCGGTATCATTGC
AGCACTGGGGCCAGATGGTAAGCCCTCCCGTATCGTAGTTATCTACACGACGGGGAGTCAGGCAACTATGGATGAACGAAATAGACAG
ATCGCTGAGATAGGTGCCTCACTGATTAAGCATTGGTAACTGTCAGACCAAGTTTACTCATATATACTTTAGATTGATTTAAAACTTC
ATTTTTAATTTAAAAGGATCTAGGTGAAGATCCTTTTTGATAATCTCATGACCAAAATCCCTTAACGTGAGTTTTCGTTCCACTGAGC
GTCAGACCCCGTAGAAAAGATCAAAGGATCTTCTTGAGATCCTTTTTTTCTGCGCGTAATCTGCTGCTTGCAAACAAAAAAACCACCG
CTACCAGCGGTGGTTTGTTTGCCGGATCAAGAGCTACCAACTCTTTTTCCGAAGGTAACTGGCTTCAGCAGAGCGCAGATACCAAATA
CTGTTCTTCTAGTGTAGCCGTAGTTAGGCCACCACTTCAAGAACTCTGTAGCACCGCCTACATACCTCGCTCTGCTAATCCTGTTACC
AGTGGCTGCTGCCAGTGGCGATAAGTCGTGTCTTACCGGGTTGGACTCAAGACGATAGTTACCGGATAAGGCGCAGCGGTCGGGCTGA
ACGGGGGGTTCGTGCACACAGCCCAGCTTGGAGCGAACGACCTACACCGAACTGAGATACCTACAGCGTGAGCTATGAGAAAGCGCCA
CGCTTCCCGAAGGGAGAAAGGCGGACAGGTATCCGGTAAGCGGCAGGGTCGGAACAGGAGAGCGCACGAGGGAGCTTCCAGGGGGAAA
CGCCTGGTATCTTTATAGTCCTGTCGGGTTTCGCCACCTCTGACTTGAGCGTCGATTTTTGTGATGCTCGTCAGGGGGGCGGAGCCTA
TGGAAAAACGCCAGCAACGCGGCCTTTTTACGGTTCCTGGCCTTTTGCTGGCCTTTTGCTCACATGTTCTTTCCTGCGTTATCCCCTG
ATTCTGTGGATAACCGTATTACCGCCTTTGAGTGAGCTGATACCGCTCGCCGCAGCCGAACGACCGAGCGCAGCGAGTCAGTGAGCGA
GGAAGCGGAAGAGCGCCCAATACGCAAACCGCCTCTCCCCGCGCGTTGGCCGATTCATTAATGCAG
```
